# Use of Cranial Ultrasound Prior to the Start of Therapeutic Hypothermia for Newborn Encephalopathy

**DOI:** 10.7759/cureus.37681

**Published:** 2023-04-17

**Authors:** Jaber Alfaifi

**Affiliations:** 1 Pediatrics, College of Medicine, University of Bisha, Bisha, SAU

**Keywords:** hypoxic-ischemic encephalopathy, newborn encephalopathy, therapeutic hypothermia, neonates, cranial ultrasound

## Abstract

For a precise diagnosis of infant hypoxic-ischemic encephalopathy (HIE), neuroimaging is required. The nature and time of the brain injury, the imaging modalities used, and the timing of their application all affect the therapeutic usefulness of neuroimaging in neonatal HIE. Most neonatal intensive care units (NICUs) across the world have access to cranial ultrasound (cUS), a safe, low-cost piece of technology that may be used at the patient's bedside. Infants undergoing active therapeutic hypothermia (TH) must undergo a cUS to be screened for intracranial hemorrhage (ICH), according to the clinical practice guidelines. The guidelines advise brain cUS on days 4 and 10-14 of life after hypothermia therapy is finished in order to thoroughly assess the nature and severity of any brain impairment. Early cUS is meant to rule out major ICH, which is listed in the local guideline for TH as a relative exclusion factor. This study questions whether cUS should be a required screening method before the start of TH.

## Introduction and background

Neuroimaging in newborns is necessary for the accurate diagnosis of hypoxic-ischemic encephalopathy (HIE) [1,2]. The type and timing of the brain injury, the imaging technology utilized, and the timing of its administration all have an impact on the clinical value of neuroimaging in newborn HIE. Cranial ultrasound (cUS), a safe, affordable piece of technology that may be utilized at the patient's bedside, is available in the majority of neonatal intensive care units (NICUs) around the world [3]. The use of cUS to identify brain injury caused by HIE has been studied. Acute, subacute, and chronic HIE characteristics affecting both gray and white matter can be found using cUS [3]. A recently proposed, validated, and associated cUS score system has been shown to predict future neurodevelopmental outcomes [4].

Magnetic resonance imaging (MRI) can detect brain injury in infants within the first few days after HIE by primarily using diffusion-weighted imaging (DWI) and magnetic resonance spectroscopy (MRS) [5,6]. Both the watershed injury and the cerebral-deep nuclear gray matter injury, which are both linked to HIE, have been identified by MRI and have been demonstrated to have prognostic value [7,8]. MRI data have been used as biomarkers for therapeutic neuroprotective investigations in infants because they can forecast future neurodevelopmental outcomes [9-13].

The Neonatal Neurocritical Care Special Interest Group (NNCC-SIG) recently conducted a survey that revealed considerable differences in the effectiveness of cUS in infants with neonatal encephalopathy (NE) [11]. Given that the majority of radiological abnormalities occur over longer time periods than the restricted six hours required for commencing therapeutic hypothermia (TH), neuroimaging is unlikely to be a reliable biomarker when employed as a screening tool before TH [12]. Notwithstanding this restriction, many hospitals continue to perform cUS on newborns to check for congenital anomalies, HIE mimics, antenatally acquired injuries, and, most importantly, major intracranial hemorrhage (ICH), which may be a relative contraindication for TH.

## Review

Materials

According to the practice guidelines, the present review article is planned to evaluate the most recent recommendation of cUS for ICH in the infants who received TH. The guidelines propose the use of brain cUS on days 4 and 10-14 of life after hypothermia therapy is finished in order to completely assess the nature and severity of any brain impairment [14]. Early cUS is intended to rule out central nervous system malformations and serious ICH, which is included as a relative exclusion factor for TH in its local guideline. Nonetheless, TH is not put off until the conclusion of cUS or the decision. In this study, a comparison was done between cUS at the first day and MRI at the fourth day of life.

The linear probe of cUS was utilized for the examination of the baby's head and was performed by the transducer of GE LOGIQTME [9,10]. The cUS protocol typically produces eight sagittal images (including midline, right and left parasagittal, and occipital horn view from the posterior fontanelle), six coronal images (from anterior to posterior), coronal clips (from frontal through parietal-temporal), and an axial view of the posterior fossa from the mastoid region [9]. This study used a more restricted procedure that involved checking the anterior fontanelle for substantial ICH. Selection criteria for TH were patients with moderate-to-severe HIE who were born at 35 weeks of gestation with birth weight greater than or equal to 1800 g and without any relative contraindications (e.g. uncontrolled pulmonary hypertension, arrhythmia requiring treatment (not sinus bradycardia), critical bleeding or coagulopathy, major congenital abnormalities). TH should begin within six hours of delivery and be maintained for 72 hours, with a target core temperature (esophageal or rectal) maintained at 33°C-34°C for 72 hours [4-6]. However, gentle rewarming should be performed slowly, and the core temperature should rise no more than 0.5°C/hour to return to normal [4-6]. Patients typically display symptoms or indications of severe fetal compromise, acute perinatal brain injuries, and postnatal clinical encephalopathy. To help in the identification of moderate-to-severe encephalopathy, amplitude-integrated electroencephalography (aEEG) was used for 20 minutes during the Cool-Cap trial and 30 minutes during the whole-body hypothermia for newborn encephalopathy (TOBY) experiment [7,8].

Methods for TH

Whole-body cooling (WBC) was used to reduce body temperature in accordance with American Heart Association (AHA)/International Liaison Committee on Resuscitation guidelines, data from systematic reviews, and meta-analyses that stimulate TH [1,2,11]. Recently available evidence revealed no appreciable differences between the two methods in terms of adverse effects, 12-month neuromotor development, or mortality rates [12,13,15-17]. The Task Force, therefore, suggested that newborn patients with HIE be treated with either WBC or selective head cooling (SHC). SHC currently employs the Olympic Medical Cool-Cap System (Olympic Medical, Seattle, WA, USA). Compared to WBC devices, it is more expensive and sophisticated, and radiant warmer changes must be done by hand [18-20].

Based on the 2010 American Academy of Pediatrics/AHA Guidelines for Neonatal Resuscitation, the Task Force advises practitioners to prevent unintentional hyperthermia in neonates as they wait for transfer. If they are placed in front of a radiant heater, it should be in the servo control mode and adjusted to a temperature that is slightly below the required 36.5°C or, at the referring hospital's suggestion, it should be turned off [19]. In order to prevent hyperthermia or overcooling, the Task Force recommends that the incubator be set at 25°C during transport and that the infant's temperature be checked often. The target core temperature during transport should be maintained at 33°C-34°C.

Temperature maintenance and rewarming

For patients receiving WBC, it has been advised by many organizations that the goal temperature is maintained at 33°C-34°C for 72 hours and that the temperature (rectal) for individuals having SHC should be kept between 34°C and 35°C for 72 hours [21]. Rewarming was done gradually, with a core temperature increase of no more than 0.5°C/hour. According to certain theories, quick rewarming or hypotension brought on by peripheral vasodilation may result in electrolyte imbalances (hypoglycemia and hyperkalemia); however, no randomized controlled trials have shown that these effects exist [22]. To prevent overcooling or hyperthermia, the temperature should be recorded and measured consistently during the cooling and rewarming periods.

Monitoring and management of neonatal encephalopathy

Electrographic seizures, electrographic status epilepticus, and 40% electrographic delirium were observed in patients with HIE who received TH and suffered from mild seizures. Term HIE infants were studied in an observational cohort study conducted across three centers with ongoing video EEG monitoring. Nine neonates (10%) had electrographically confirmed status epilepticus, for a total of 43 neonates (48%) with electrographically diagnosed seizures. It is currently unclear whether newborn convulsions have any additional negative effects on newborns with underlying encephalopathy [23,24].

In the setting of TH, seizures do in fact remain a risk factor for brain injury, especially in people with status epilepticus or multifocal onset seizures who require a number of medications. Given that asphyxiated newborns frequently experience seizures, particularly during the first two days of life, the Task Force advises that seizure activity should be monitored crucially; therefore, it is advised to use an EEG or continuous video EEG to monitor seizures [25]. Because phenobarbital alone can successfully treat newborn seizures in 50% of instances, in 36% of cases it may be necessary to take two or more antiepileptic drugs [26]. When seeking seizure treatment, the Task Force suggests consulting a pediatric neurologist, particularly if extra or multiple medications are required [26].

Cranial US/neuroimaging

**Figure 1:**
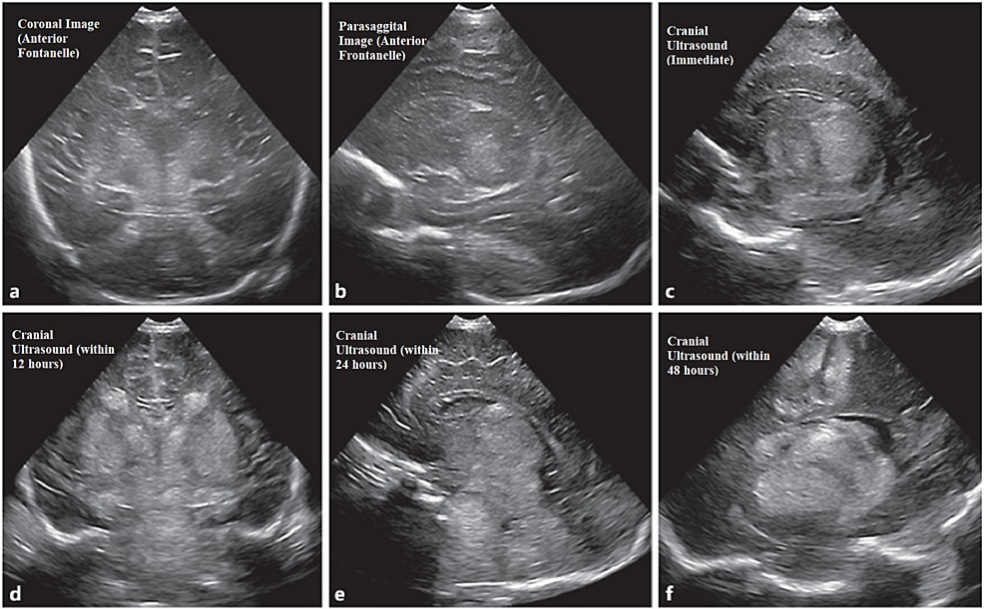
cUS with distinctive imaging pattern in HIE. (a) Full-term neonate with coronal image through AF; (b) full-term neonate with parasagittal image through AF; (c) cUS showing ischemic injury marked by bilateral symmetric increased echogenicity in thalami shows a central pattern of injury; (d) ventricles are slit-like due to edema from the central structures within (d) 12 hours of cUS, (e) 24 hours of cUS, and (f) 48 hours of cUS. AF: anterior fontanelle; cUS: cranial ultrasound; HIE: hypoxic-ischemic encephalopathy.

**Figure 2:**
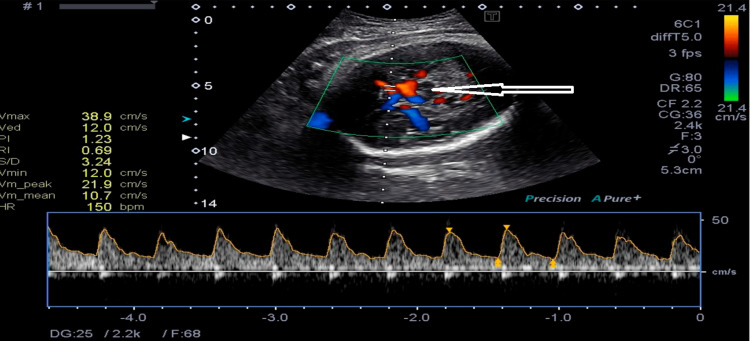
Doppler imaging of cerebral blood flow through MCA (arrow) showing changes at 33 weeks of GA in HIE. MCA: middle cerebral artery; HIE: hypoxic-ischemic encephalopathy; GA: gestational age.

**Figure 3:**
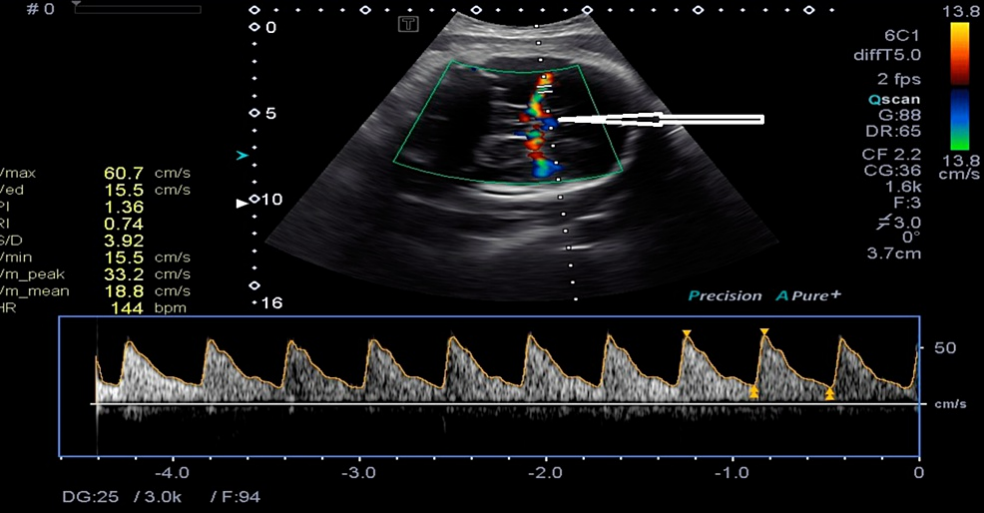
Doppler imaging of cerebral blood flow through MCA (arrow) showing marked changes at 37 weeks of GA in HIE. MCA: middle cerebral artery; HIE: hypoxic-ischemic encephalopathy; GA: gestational age.

The observed sensitivity for lesions in term babies with HIE in the early days of cUS anomalies was good when imaging was done after the first week of life (Figure 1) [25]. These first-generation real-time sector scanners were able to identify cerebral bleeding, extra-axial fluid collections, changes in ventricular size, and periventricular echogenicity utilizing a 5 MHz mechanical sector transducer. Moreover, the increased echogenicity of thalami was found [26]. Comparing the two imaging modalities, MRI had a superior occurrence of anomalies in the basal ganglia/thalami and periventricular white matter. However, cUS scans detected all infants who would have a bad prognosis [27]. Changes in cerebral blood flow were seen using Doppler techniques (Figures 2, 3).

It is practical to assess brain injury in newborns who are both full-term and preterm using cUS. The resistance index (RI) of Pourcelot was 0.55 at 24 and 62 hours after birth, regarded as a powerful predictor of a very poor result and mortality before cooling therapy [27,28]. Thirty-four full-term infants with HIE were investigated to determine the sensitivity and specificity of cUS. Within six hours of birth, the sensitivity and specificity of cUS were found to be 42% and 60%, respectively. [23,28].

Another retrospective study was conducted by William Sanislow on 108 infants and showed a discrepancy between initial cUS and follow-up brain MRI findings [29]. cUS can detect embryonic brain damage and congenital structural abnormalities of the brain. Moreover, it can detect anomalies brought on by different forms of newborn encephalopathies, such as hypoplastic corpus callosum, germinal cysts, mitochondrial or peroxisomal disorders, extreme white matter echogenicity, often known as "HIE-mimics," and molybdenum cofactor deficiency, which are all examples of conditions that can cause this condition [25,29]. Neonatal encephalopathy screening with cUS has been advocated for newborns admitted to NICUs. It does indicate the onset of neonatal encephalopathy when there is augmented echogenicity in the white matter at birth. The second half will mark the onset of central gray nuclei and PAIS abnormalities.

Adjuvant therapy for TH

Many factors underlie newborn HIE. In newborns with HIE, TH increases survival and lowers the rate of disability, but death or disability still occurs. According to statistics from significant clinical trials, the rate is still high (40%-50%). Thus, there is a critical need for additional therapy to enhance HIE results [23,27]. 

Erythropoietin, topiramate (TPM), allopurinol, magnesium sulfate, xenon, melatonin, and stem cell therapy are among the adjuvant treatments being studied for use with TH. Intravenous injections of erythropoietin (1000 U/kg/dose) can increase neurogenesis, reduce oxygen free radicals and the inflammatory response to hypoxia, and promote the expression of anti-apoptotic genes relative to pro-apoptotic genes. According to the information that was available, darbepoetin combined with hypothermia had a safety profile equivalent to that of a placebo and had pharmacokinetics suitable for weekly administration [28].

TPM multicenter randomized controlled trial observed a decrease in the prevalence of epilepsy in newborns co-treated with TPM. An inert noble gas known as xenon is employed for its anesthetic characteristics, which are mediated via competitive binding at the N-methyl-D-aspartate (NMDA) glutamate receptor's glycine binding site. Xenon has the ability to lessen excitotoxicity while also triggering pro-survival kinases including p-Akt (protein kinase) and the anti-apoptotic factor [29]. It has been suggested that the NMDA receptor antagonist magnesium sulfate (MgSO_4_) can effectively protect the brain. The effectiveness of magnesium therapy in preventing mortality or moderate-to-severe impairment in babies with HIE has not been adequately studied [30].

## Conclusions

cUS is convenient to evaluate brain injury in both full-term and preterm newborn infants. Although MRI has a superior ability to detect anomalies in the basal ganglia/thalami and periventricular white matter, cUS scans can detect infants who would have a bad prognosis. The observed sensitivity of cUS for lesions in term babies with HIE is appreciable, particularly when imaging is done after the first week of life. Moreover, cUS is beneficial in figuring out the etiology of encephalopathy. Advances in the cUS have substantially progressed its detection abilities of anomalies, and through repeated evaluations, it is now able to detect the pattern, timing, and severity of injury.

## References

[1] Edwards AD, Brocklehurst P, Gunn AJ (2010). Neurological outcomes at 18 months of age after moderate hypothermia for perinatal hypoxic ischaemic encephalopathy: synthesis and meta-analysis of trial data. BMJ.

[2] Jacobs SE, Berg M, Hunt R, Tarnow-Mordi WO, Inder TE, Davis PG (2013). Cooling for newborns with hypoxic ischaemic encephalopathy. Cochrane Database Syst Rev.

[3] Kattwinkel J, Perlman JM, Aziz K (2010). Part 15: neonatal resuscitation: 2010 American Heart Association Guidelines for Cardiopulmonary Resuscitation and Emergency Cardiovascular Care. Circulation.

[4] Chiang MC, Lien R, Chu SM (2016). Serum lactate, brain magnetic resonance imaging and outcome of neonatal hypoxic ischemic encephalopathy after therapeutic hypothermia. Pediatr Neonatol.

[5] Wyckoff MH, Aziz K, Escobedo MB (2015). Part 13: neonatal resuscitation: 2015 American Heart Association Guidelines Update for Cardiopulmonary Resuscitation and Emergency Cardiovascular Care. Circulation.

[6] Shankaran S, Laptook AR, Ehrenkranz RA (2005). Whole-body hypothermia for neonates with hypoxic-ischemic encephalopathy. N Engl J Med.

[7] Gluckman PD, Wyatt JS, Azzopardi D (2005). Selective head cooling with mild systemic hypothermia after neonatal encephalopathy: multicentre randomised trial. Lancet.

[8] Azzopardi DV, Strohm B, Edwards AD (2009). Moderate hypothermia to treat perinatal asphyxial encephalopathy. N Engl J Med.

[9] Simbruner G, Mittal RA, Rohlmann F (2010). Systemic hypothermia after neonatal encephalopathy: outcomes of neo.nEURO.network RCT. Pediatrics.

[10] Jacobs SE, Morley CJ, Inder TE (2011). Whole-body hypothermia for term and near-term newborns with hypoxic-ischemic encephalopathy: a randomized controlled trial. Arch Pediatr Adolesc Med.

[11] Tagin MA, Woolcott CG, Vincer MJ, Whyte RK, Stinson DA (2012). Hypothermia for neonatal hypoxic ischemic encephalopathy: an updated systematic review and meta-analysis. Arch Pediatr Adolesc Med.

[12] Celik Y, Atıcı A, Gulası S, Okuyaz C, Makharoblıdze K, Sungur MA (2016). Comparison of selective head cooling versus whole-body cooling. Pediatr Int.

[13] Robertson NJ, Kendall GS, Thayyil S (2010). Techniques for therapeutic hypothermia during transport and in hospital for perinatal asphyxial encephalopathy. Semin Fetal Neonatal Med.

[14] Salas J, Tekes A, Hwang M, Northington FJ, Huisman TAGM (2018). Head ultrasound in neonatal hypoxic-ischemic injury and its mimickers for clinicians: a review of the patterns of injury and the evolution of findings over time. Neonatology.

[15] Shankaran S, Laptook A, Wright LL (2002). Whole-body hypothermia for neonatal encephalopathy: animal observations as a basis for a randomized, controlled pilot study in term infants. Pediatrics.

[16] Pfister RH, Bingham P, Edwards EM (2012). The Vermont Oxford Neonatal Encephalopathy Registry: rationale, methods, and initial results. BMC Pediatr.

[17] Fairchild K, Sokora D, Scott J, Zanelli S (2010). Therapeutic hypothermia on neonatal transport: 4-year experience in a single NICU. J Perinatol.

[18] Chaudhary R, Farrer K, Broster S, McRitchie L, Austin T (2013). Active versus passive cooling during neonatal transport. Pediatrics.

[19] Goenka A, Yozawitz E, Gomes WA, Nafday SM (2020). Selective head versus whole body cooling treatment of hypoxic-ischemic encephalopathy: comparison of electroencephalogram and magnetic resonance imaging findings. Am J Perinatol.

[20] Archer LN, Levene MI, Evans DH (2016). Cerebral artery Doppler ultrasonography for prediction of outcome after perinatal asphyxia. Lancet.

[21] Eken P, Toet MC, Groenendaal F, de Vries LS (1995). Predictive value of early neuroimaging, pulsed Doppler and neurophysiology in full term infants with hypoxic-ischaemic encephalopathy. Arch Dis Child Fetal Neonatal Ed.

[22] Eken P, Jansen GH, Groenendaal F, Rademaker KJ, de Vries LS (1994). Intracranial lesions in the fullterm infant with hypoxic ischaemic encephalopathy: ultrasound and autopsy correlation. Neuropediatrics.

[23] Epelman M, Daneman A, Halliday W, Whyte H, Blaser SI (2012). Abnormal corpus callosum in neonates after hypoxic-ischemic injury. Pediatr Radiol.

[24] Dinan D, Daneman A, Guimaraes CV, Chauvin NA, Victoria T, Epelman M (2014). Easily overlooked sonographic findings in the evaluation of neonatal encephalopathy: lessons learned from magnetic resonance imaging. Semin Ultrasound CT MR.

[25] de Vries LS, Cowan FM (2009). Evolving understanding of hypoxic-ischemic encephalopathy in the term infant. Semin Pediatr Neurol.

[26] De Vries LS, Smet M, Goemans N, Wilms G, Devlieger H, Casaer P (1992). Unilateral thalamic haemorrhage in the pre-term and full-term newborn. Neuropediatrics.

[27] Govaert P, Achten E, Vanhaesebrouck P, De Praeter C, Van Damme J (1992). Deep cerebral venous thrombosis in thalamo-ventricular hemorrhage of the term newborn. Pediatr Radiol.

[28] de Vries LS, Groenendaal F, Eken P, van Haastert IC, Rademaker KJ, Meiners LC (1997). Infarcts in the vascular distribution of the middle cerebral artery in preterm and fullterm infants. Neuropediatrics.

[29] Cowan F, Mercuri E, Groenendaal F, Bassi L, Ricci D, Rutherford M, de Vries L (2005). Does cranial ultrasound imaging identify arterial cerebral infarction in term neonates?. Arch Dis Child Fetal Neonatal Ed.

[30] Barkovich AJ, Truwit CL (1990). Brain damage from perinatal asphyxia: correlation of MR findings with gestational age. AJNR Am J Neuroradiol.

